# Views from patients, students, and preceptors about the ethics of student-run free clinics

**DOI:** 10.1007/s10900-024-01438-2

**Published:** 2025-01-20

**Authors:** Isabella Li, Caroline R. Morehouse, Claire M. Moore, Gabriela Esnaola, Andres Aguirre, Annette Rid, Christine Grady

**Affiliations:** 1 Department of Bioethics, The Clinical Center, National Institutes of Health, Building 10, Room 1C118, Bethesda, 20892 USA; 2 Yale University School of Medicine, New Haven, CT USA; 3 Yale College, New Haven, CT USA

## Introduction

At roughly three-quarters of U.S. medical schools, health professional students run student-run free clinics (SRFCs) to provide free or reduced-cost care—including, for example, primary care services, medications, and social services—to their local communities [1, 2]. Typically, student volunteers engage with patients, then consult with licensed preceptor volunteers to design treatment plans [3]. Patients at SRFCs often face one or multiple forms of structural disadvantage that limit their access to healthcare, including being unhoused, uninsured, or of low socioeconomic status, and they tend to belong to racially or ethnically minoritized communities [2, 4]. 

In surveys and a limited number of qualitative studies, patients reported high levels of satisfaction with SRFC care [5–8]. Single SRFCs have described patient outcomes that are equivalent to or better than those at other clinics, as well as reduced utilization of emergency departments [9–11]. Student volunteers report gaining clinical skills, awareness of interprofessional collaboration and health systems, knowledge of working with underserved populations, and an increased willingness to specialize in primary care [12–15]. 

Despite these benefits, SRFCs have limitations. SRFC patients have reported negative experiences such as long wait times, and in non-SRFC settings, patients have indicated discomfort with student involvement [5, 8, 16, 17]. Meanwhile, some SRFC student volunteers have reported feeling unprepared and note clinic resource shortages, especially for complex cases [18–20]. 

Ethical discussions of SRFCs touch on similar topics [20–23]. Some note that patients benefit from receiving otherwise inaccessible care while students learn clinical skills and gain opportunities for altruistic engagement [2, 21]. Others, however, have raised concerns. Relative to other providers, SRFCs may offer patients lower quality care due to resource shortages and students’ inexperience [20, 23]. In addition, ethicists have suggested that students may be discouraged from working in free clinic settings in the future due to stressful SRFC experiences, or worry that students may develop the belief that it is acceptable to provide low-income patients with substandard care [21, 23]. As such, SRFCs may raise concerns about exploitation, in which students take advantage of patients’ limited options to benefit their own professional development [20, 22, 23]. Finally, some ethicists have criticized SRFCs as “stopgap measures” that harm patients by detracting attention from underlying causes of health inequity in the U.S [22]. 

Detailed descriptions of how SRFC stakeholders perceive and experience the benefits, limitations, and ethical issues associated with SRFCs remain scarce in the literature. Moreover, to our knowledge, there are no studies that juxtapose the perspectives of patients, student volunteers and preceptors from the same SRFC. Our study aimed to fill this gap in the literature by documenting patient, student and preceptor perspectives at two urban SRFCs in the U.S. All interviewed students and preceptors in these clinics were volunteers, henceforth we refer to them as “students” and “preceptors.” We describe the full range of perceived benefits, limitations, and ethical issues of SRFCs reported by our respondents. We then draw on these data to suggest recommendations for how SRFCs might address the identified ethical issues. Throughout, we use the term “ethical issues” interchangeably with related terms, such as “ethical challenges” or “ethical concerns,” to denote areas of SRFC practice that require ethical attention.

## Methods

### Ethics

We designed recruitment and interview approaches in consultation with clinic leadership to be minimally disruptive to clinic workflow and patient well-being. Because we did not record identifiable information, the study was deemed exempt from IRB review by the NIH IRB, as well as IRBs at the two sites. To further protect confidentiality, both clinics requested that details about their setting, operation, and patient population remain anonymous or be reported in aggregate.

Table 1 provides a summary of the setting and methods used in this study, including relevant methodological differences between individual clinics.

**Table 1:** Summary of setting, recruitment methods, data collection methods, and data analysis methods in qualitative study of two student-run free clinics (SRFCs), 2023

	SRFC 1	SRFC 2
*Setting*		
Location	East Coast city	
Frequency	Open once a week	
Patient population	Unhoused and/or uninsured people in local community	
Services offered	Medical (e.g. primary care, behavioral health, reproductive health, tests and screenings, medications, referrals to other providers) Non-medical (e.g. social services, free hygiene products, health education, legal assistance, childcare)	
Training	Clinic-wide presentation on best practices (e.g. clinic purpose/philosophy/organizational structure, walkthrough of visits, notes on patient privacy/HIPAA) as well as varying team-specific training (e.g. shadowing more experienced students, reading a written handbook)	
Supervision	The least experienced students provide non-medical services (e.g. translation, social services), or, when involved in providing medical services, see patients under the guidance of more experienced students. Students ultimately consult with preceptors about each case	
*Participant recruitment*		
Patients	Students volunteering in that day’s clinic approached patients—sampled purposively based on known utilization of clinic services—during visit to assess interest in being interviewed. Eligibility criteria: Over 18, visited clinic at least once.	
Students	Clinic leader circulated information. Eligible students contacted clinic leader. Eligibility criteria: Had volunteered at the clinic at least 3 times.	Clinic leader circulated information. Eligible students contacted research team directly. Eligibility criteria: Had volunteered at the clinic at least 3 times.
Preceptors	Clinic leader informed research team of consistent preceptors. Research team emailed preceptors and scheduled interview. Eligibility criteria: Had volunteered at the clinic at least 3 times.	
*Data collection*		
Patients	Researchers (alone or in pairs) conducted individual interviews with patients waiting for other services. 12 patients total. Compensation: $25 grocery store gift card. Demographic information collected via paper survey.	Researchers (one member leading, 1–2 others asking follow-up questions) conducted focus groups with 3–4 patients on the day of their appointment. 11 patients total. Compensation: $50 grocery store gift card. Demographic information collected via paper survey.
Students	Researchers (one member leading, 1–2 others asking follow-up questions) conducted focus groups. One focus group in-person with 8 participants, one focus group on Zoom with 10 participants. 18 participants total. Compensation: Snacks and refreshments. Demographic information collected via paper or email survey.	Researchers (one member leading, 1–2 others asking follow-up questions) conducted focus groups. Four groups take place over Microsoft Teams, with 2–4 participants each. 14 participants total. Compensation: $10 Amazon gift cards. Demographic information collected via email survey.
Preceptors	Researchers (alone or in pairs) conducted individual interviews on Microsoft Teams. 3 preceptors interviewed. No demographic information collected.	Researchers (alone or in pairs) conducted individual interviews on Microsoft Teams. 4 preceptors interviewed. No demographic information collected.
*Data analysis*		
Interviews/focus groups	Professional services transcribed and (if applicable) translated Spanish interviews. Abductive analysis to generate coding scheme. Two independent coders for each interview.	
Document review	Informal analysis of public facing websites, annual reports, and training materials provided by clinic leadership.	

### Study setting

Patients, students, and preceptor were interviewed from two university affiliated SRFCs in two cities on the U.S. East Coast. Despite differences in operation, both clinics aim to provide some healthcare services to patients facing structural disadvantage in their local communities. Both operate once a week and offer a range of medical and non-medical services. Volunteers at both clinics received training through clinic-wide presentations on best practices, as well as varying role-specific training. Both clinics have similar supervisory structures, in which less experienced students work with more experienced students and consult with preceptors on all medical activities.

### Participant recruitment and sampling

This study used non-probability-based sampling. During appointments, students volunteering at the clinic introduced the study to patients, selected through purposive sampling based on their experiences receiving clinic services. Clinic leaders circulated an invitation from the research team to students, and interested students contacted a clinic leader or the research team. Our team directly emailed preceptors whom clinic leadership identified to introduce the study and schedule interviews. We informed all participants of the confidentiality of their responses.

### Data collection

Interview and focus group questions for patients, students, and preceptors centered on participants’ perceptions of their experiences at the clinic, comprehensiveness of care, provider-patient interactions, student involvement, and comparisons with other clinical settings (see Appendix A). We piloted the interview guide with 3 NIH Clinical Center patients and 4 current and former students who had volunteered at student-run free clinics.

Researchers, alone or in pairs along with a Spanish interpreter if necessary, conducted semi-structured individual interviews or 3–4 person focus groups with patients on the day of their clinic appointments. Patients received $25–50 grocery gift cards as compensation. IL and a secondary facilitator conducted focus groups with a range of 2–10 students, either in-person or online outside of clinic hours. Student participants were compensated with refreshments or $10 Amazon gift cards. We collected demographic information via paper or email surveys for patients and students. With preceptors, semi-structured interviews were conducted, either alone or in pairs, on Microsoft Teams. We did not collect preceptor demographic information.

### Data analysis

Professional services transcribed the interviews and, when applicable, translated Spanish interviews. IL generated the coding scheme through initial transcript review using an abductive analysis technique, integrating concepts from existing literature with perspectives drawn directly from the data [24]. IL, CRM, and CG reviewed, discussed, and modified the coding scheme. Using NVivo (version 14.23.2; Lumivero; Denver, Colorado), two researchers independently coded each interview, with IL coding all and CRM or CG coding a subset. We resolved disagreements by discussion.

An informal review of clinic documents, including public facing websites, annual reports, and training materials provided to us by clinic leadership, supplemented the analysis.

## Results

### Participant characteristics

Interviewed patients (*N* = 23) were mostly women of color with varied housing status, insurance status, income, and English-speaking ability (Table 2). Students (*N* = 32) were primarily medical students with a few undergraduate students and students from other health professional schools (Table 3). One-third had participated in clinical rotations. All preceptor participants (*N* = 7) were licensed providers and ranged in career stage from residency to retirement. They held experience in various clinical specialties and settings.

**Table 2:** Characteristics of patients interviewed at two student-run free clinics, 2023

	Responses (*N* = 21)^a^
**Gender**	
Woman	20
Man	1
**Self-reported race/ethnicity** ^**b**^	
Black or African American	7
Hispanic, Latino, or Spanish origin	10
White	2
Multiple	1
**Insurance status**	
Insured	10
Uninsured	11
**Annual household income** ^**c**^	
Range	$0-$44,725
Median	$1,400
Average	$10,500
Mode	$0 (*N* = 7)
**Housing status**	
Housed	12
Unhoused	9
**Primary language**	
English	10
Spanish	11

a. Missing demographic data from 2 of 23 total patients interviewed due to error in data collection

b. 1 participant did not answer

c. 1 participant was unsure, 1 participant did not answer

**Table 3:** Characteristics of students interviewed at two student-run free clinics, 2023

	Responses (*N* = 31)^a^
**Number of shifts volunteered** ^b^	
4	4
5–10	4
11–20	5
> 20	18
**School**	
Undergraduate	2
Medical	24
Nursing	3
Public Health	2^c^
Physician Assistant	1^c^
**Year in school** ^**d**^	
1	1
2	20
3	5
Research gap year	2
4	2
**Clinical rotation experience**	
Yes	10
No	21

a. Interviewed 32 students, 1 student did not complete the post-interview survey

b. Some students participated in various roles and/or departments. Here, we report their overall total number of shifts volunteered, except for one student, who only reported the number of shifts they had volunteered in their current role

c. One student in a joint PA/MPH program

d. One student did not answer

### Patient care in resource-limited settings

Patients, students, and preceptors valued the medical and social services SRFCs provided to patients with limited financial and social resources, but also expressed frustration at challenges inherent to patients’ circumstances and the clinics’ scope, organization, and capacity.

#### Benefits

Most patients, students, and preceptors expressed satisfaction with their SRFC’s basic medical services including general physical examinations, care for minor infections or pain, blood pressure management, and access to contraception. Several students described their SRFC as a “bridge” referring patients to outside providers for more consistent primary care or specialized needs—sometimes through connections with providers at their educational institution.

Patients described SRFCs as more logistically and financially accessible than other medical options. Some valued the SRFC for its convenient location and appointment times (Table 4). Uninsured patients valued the SRFC’s free care over expensive alternatives. Patients contrasted one SRFC with the emergency room, which they found financially infeasible (Table 4). This SRFC, patients, students, and preceptors noted, was even more financially accessible than a local federally qualified health center (FQHC), which used a sliding scale payment system and did not provide some of the SRFC’s services such as transportation assistance.

**Table 4:** Illustrative quotations of benefits and limitations of student-run free clinics as identified by patients, students, and preceptors at two sites, 2023

Theme	Subtheme	Illustrative quote
*Patient care in resource-limited settings*		
Benefits	SRFC is more logistically accessible for patients than other providers	With me working, I can’t call out for 60 days, so I can’t make doctor’s appointments just because a cough comes up. So that’s good to know that every Wednesday you can come talk to somebody and make sure if you got to go to the hospital or if they can just tell me, “Let’s wait it out and see what happens, and we’ll see you next week.” (Patient)
	SRFC is more financially accessible for patients than other providers	Patient 1: Since [at the hospital emergency room] they know we don’t have insurance, they almost let me die there… And my bill, just for checking me, the first one was 1,500 and then for the exams and everything, it was 4,500 and in total they charged me about 8,000 dollars… Patient 2: We who do not have insurance, in order not to go to an emergency [room], we let ourselves die at home. Or what do we do? I am already afraid of emergencies. I wait to come here. Patient 1: That is why this clinic [the SRFC] is so important to us.
	SRFC offers patients non-medical services	Pampers are a must. Wipes are a must. And it’s crazy, because [the SRFC] is the only reliable source that we have… But the little things that they give us like shampoos, conditioners, tampons, underwear, things like that… if I’m trying to save money to move somewhere, those little things are helpful. (Patient)
Limitations	SRFC can divert from other, better resourced providers	We’re actually giving less [than an FQHC], but they don’t perceive it that way because we’re doing the day-to-day funding of their medications, transportation, we’re calling them, reminding them of appointments, we’re following up. (Preceptor)
	SRFC may be unable to provide continuous care, especially for chronic or ongoing conditions	Someone might come in and they have a history of COPD and they are interested in maybe wanting some steroids… But when you talk with them more, this is their third course of steroids this month, or things like that, that I’m, in the grand scheme of things, not comfortable with following through with that plan. I maybe have that bigger picture idea of, this is 100% one of those patients that we need to focus on engaging them with care as opposed to just treating this acute problem. Because I actually think that in treating this acute problem, we could be doing them more damage by just continuously prescribing them ongoing steroids, for example, that could be giving them other side effects and raising other concerns. (Preceptor)
	SRFC’s care is limited; available alternatives for patients are also limited	There was one patient who had a lot of chronic pain and had a very complex history, to the point where she had pretty much exhausted all the resources we had at [the SRFC], in terms of medications we could give her, because we can’t prescribe narcotics. And so, we were like, “The pain that you have and the amount of support that you need is kind of above our capability, so I think it might be better for you to seek care elsewhere, where they might have more support.”… She ended up not being able to follow up with making an appointment somewhere else, and she also worried about the cost, and all these other things… In the end we took her back. It was better to have some care than no care at all. (Student)
*Student involvement in patient care*		
Benefits	Students have better interactions with patients than licensed providers do	I have never felt uncomfortable with them [students]. I think that there are young people here, everyone who serves is young people, and in my opinion that has a lot of influence. Sometimes people who are already older, maybe stress, tiredness, they had a problem at home, whatever, that brings them stressed to work… but in this clinic, there are young people and they come active, with a fresh mind, with the willingness to care for people well. (Patient)
	Patients say they rely on the supervision that students receive and feel comfort knowing that the supervisor is available to them	I’m fine with [being seen by students], as long as, if I don’t understand, then I want somebody who knows more… if I’m saying something and I don’t understand, they will bring the doctor in. But they try to explain to the best of their ability to me. But then it could be like they’re students and I’m like, “These is not no real doc.” So especially with the high blood pressure, I was worried. So I wanted somebody to actually know what was going on. (Patient)
	Students feel well-supervised	I worked it up, to the best of my knowledge, as a second-year med student. And then, I went out and spoke with the director for the day… I think he’s a fifth-year, so he’s much more experienced than I am, but he also went in and asked more questions, and then we talked to the attending, who went and asked even more questions. So it was obviously a longer process than if he had just seen a doctor, directly. But I feel confident that, by the end, all of the questions were asked and the attending was able to get all of the pertinent information… I feel like the buck never stops with me. (Student)
	Students learn to incorporate consideration of the social determinants of health in their medical practice	I think it just makes us better equipped to look at a whole person’s needs. So maybe when we’re in rotations next year, if the doctor has a recommendation for a patient, we know next steps better for how to actually make sure that their recommendation fits in with the person’s life. Is it actually feasible or not? (Student)
Limitations	Volunteering at SRFCs can take an emotional toll on students	There are limits to what you can do as a student in a free clinic, and I think that’s a pretty big challenge that we see every time we have a clinic… That can be hard managing for the patients, and that can also just be hard to deal with this emotionally as a student, because you want to do more and often you can’t. (Student)
	Heterogeneity in supervision can lead to negative patient experiences	There’s no real training before those things happen. You might quickly review in the hall with the attending if you have questions about the procedure or anything. And of course, students can always say, “I’m not equipped to do this, so I’m going to let the attending do this.” But that’s very much a self-selective process for that student… I think every [student] especially maybe has their own line of where they would be comfortable doing something that they’re not super familiar with, versus not. I think it depends a lot on the attending who’s there too. An attending might be like, “Hey, you’ve never placed an IUD before? Great. Come on in and practice.” Or they might be like, “Whoa, you’ve never placed an IUD before? I’m going to do this one.” (Student)
	Patients accept some inconveniences as a trade-off for free care	There were so many hours of waiting and waiting. But after going so much, you already process the information and you say, “Well, I’m going for an appointment at the free clinic, it takes time.” (Patient)
*Ethical acceptability*		
Benefits	Patients receive high-quality care at SRFC that may otherwise be difficult to access	Is it unfair that people who don’t have healthcare are being seen by students? I think they’re pretty lucky. The students seeing them are very, very thoughtful and listening, and that’s an important part of their care. The patients tend to feel very, very cared for. And the preceptors, we’re doing our job too, so I feel that as a way to enter the healthcare system, this is a pretty good way. (Preceptor)
	Supervision and referrals to outside providers mitigate any negative impact of student involvement	Before I joined [the SRFC], I definitely thought about this a little bit because you’re going into a community that’s already really marginalized and disadvantaged and you’re sending medical students who aren’t the most qualified. And so I kind of worried about that. But I think the way that [the SRFC] tries to address it is we’re not trying to be their main doctor. We’re trying to be a bridge to get them in to see that high quality care. We’re going to do the best that we can, and that’s why we have an actual preceptor there… And I think that a lot of our roles [such as connecting patients to social services], I feel pretty qualified to try to help with that. (Student)
Limitations	SRFCs are a stopgap measure in unjust healthcare system	I think our health system is very unjust in the fact that people that are uninsured or experiencing homelessness and don’t have the resources to be able to schedule a doctor’s appointment are going to have a very hard time getting into a doctor’s office. I think having students is a way for harm reduction as well as trying to help facilitate patients being scheduled with MDs or DOs or providers. And it’s a way to try to mitigate that barrier that a lot of these patients have to healthcare… But still, the fact that the patients are receiving care from medical students only, although very appreciated by the patients, is unfair that they don’t have proper access to a physician. (Preceptor)
	Students learn clinical skills with a vulnerable population	We are learning on a population that has limited access to other options beyond us and there’s inherent sort of potential for abuse there. (Student)

SRFC accessibility helped patients avoid more negative health outcomes. Students described cases where they identified serious concerns, such as dramatically elevated blood pressure, acute pancreatitis, or seizures, and encouraged patients to go to the hospital. One student noted that their clinic intervened in at least two cases of pre-eclampsia because patients visited the SRFC more frequently than an obstetrician.

Importantly, patients at both SRFCs frequently mentioned appreciation of non-medical services, including hygiene and toiletry products, childcare during clinic hours, legal assistance, and resources for a range of other social services (e.g. housing) (Table 4).

#### Limitations

Patients, students, and preceptors also described limitations to the continuity and comprehensiveness of care. Some limitations were reflective of challenges faced by the life circumstances of SRFC patients. Students at one SRFC mentioned the frequency of “no-shows,” which they attributed to factors such as low medical literacy and lack of transportation.

Other limitations were unrelated to patients’ circumstances. Internally, high turnover of students led to frequent disruptions in SRFC operating structure. Students and preceptors believed that challenges in coordinating care with outside providers—due to, for example, lack of access to electronic medical records and general communication breakdowns—sometimes created patient confusion. Clinics also struggled with capacity and resource availability. Some patients expressed dissatisfaction when the SRFC ran out of non-medical resources such as bus passes. With limited time and a limited number of student and preceptor volunteers, one clinic had long wait lists for new patients, and two existing patients voiced frustration about months-long waits for follow-up visits.

Patient and clinic factors often synergistically limited care. For example, one student said the SRFC did not provide certain clinically indicated medications because it could not regularly monitor patients’ blood tests. One preceptor described the SRFC’s scope as “not up to the level of a typical primary care office,” offering an example of when the limited scope of clinic’s medical services, combined with patient follow-up challenges, delayed treatment:

So, in my office, this would’ve been pretty straightforward. I would order some Lantus [long-acting insulin] once a day, my nurse would come in and make sure the person knew how to do finger sticks, would just lay out a plan, and it would be just really a few minutes. But in [the SRFC], there were these hurdles of, “Well, we don’t do insulin here, we don’t have the point-of-care testing, and so we just have to refer this person to endocrine [at an outside provider].”… This patient then went on to miss multiple subsequent visits, endocrine appointments, and still has not gotten the care that could have been started if we had these options at the clinic. (Preceptor)

Another preceptor expressed concern that the SRFC could divert patients from better resourced providers (Table 4). The preceptor believed that patients with medically complex conditions would receive better care at an FQHC, rather than a once-a-week student run clinic, as “you need to have someone… that’s regularly following you.” Patients, students, and preceptors also described situations where patients refused to see other providers—even when they could afford to—including in some cases when they needed emergency care or complex management outside of the clinic’s scope.

Students and preceptors had different ways of grappling with these limitations. Several emphasized the importance of connecting patients with other clinics for longer-term care (Table 4). One preceptor wondered whether the SRFC should focus on offering non-medical resources and “decentralize from that idea of it being a clinic,” as she was unsure whether the “medical aspect” was “adequate and right for the patients.” Still, other students and preceptors believed in the importance of the SRFC’s providing its own medical services. They emphasized working within the clinic’s means to provide some care, even when it did not fully address patients’ complex needs (Table 4).

### Student involvement in patient care

Patients, students, and preceptors reported that when well-supervised, student involvement at SRFCs generated positive interactions for patients and learning opportunities for students. However, sometimes students’ inexperience negatively impacted patient care.

#### Benefits

None of the interviewed patients indicated any surprise or confusion when asked about student involvement, contrary to some students’ concerns about patient awareness. No patients expressed discomfort with being seen by students, and many expressed support for it as part of the medical education process:

“How can they get a chance to be a doctor if they don’t have the chance to practice it?” (Patient).

In fact, many patients preferred interactions with students at SRFCs to interactions they had with licensed providers at outside facilities, perceiving students as more attentive, kind, and approachable (Table 4):

They’re very friendly, so you can say that’s the difference [between the SRFC and another local clinic]. They will sit there and talk to you, actually talk to you, and not just be like, “Oh, I’m just here to work.” They will actually talk and sit there and figure things out, help you out. (Patient)

Key to the positive experiences described by patients was proper supervision by preceptors. Both clinics had similar supervisory structures for medical visits: a junior and senior student perform a history and physical examination on a patient together, then present to a licensed preceptor who signs off on a treatment plan, most often also visiting the patient. Several patients indicated that their comfort with being seen by students hinged on students’ supervision (Table 4). Similarly, for many students, supervision offered reassurance that despite their own limited experience, patients received appropriate care (Table 4).

Students found that SRFC volunteering offered them educational experiences beyond what they would learn in other settings, including clinical rotations. They appreciated early exposure to practical skills, interprofessional collaboration, leadership, and independence. Many students mentioned that volunteering in an SRFC taught them about social justice—deeper knowledge of how social inequity impacts healthcare and skills to incorporate consideration of social determinants of health into routine medical care (Table 4). Some students appreciated that the free setting allowed them to experience more ideal models of healthcare, with longer visits and greater freedom to develop treatment plans.

#### Limitations

Volunteering placed an emotional burden on some students when patients with complex medical and social needs shared traumatic experiences—especially if students felt inadequate in addressing these needs (Table 4).

Some students felt dissatisfied with their SRFC training, which varied by SRFC and department—particularly training with passively absorbed materials (e.g. training videos) without more practical exposure (e.g. shadowing). Clinic documents revealed that neither SRFC offered ethics training, though leaders at one clinic discussed the ethics of SRFCs at a leadership-specific retreat, and at both clinics, a presentation to students providing medical services reinforced the clinic’s mission and encouraged professionalism (e.g. privacy, empathy for patients’ circumstances). One clinic’s presentation included a script for students to specify their learning role (e.g. “I am a first year nursing student…”) but it was not verbally discussed by presenters. The other advised students to “introduce yourselves” to patients.

Students also reported heterogeneity in students’ preparation, as well as preceptors’ approach to supervision (Table 4). One student observed that, because preceptors often relied on students’ self-assessment of skill level, with some quite comfortable letting inexperienced students treat patients, they “saw a lot of people who were providing healthcare services to patients without very much instruction or training” (Student). One preceptor said that, due to high patient volume, not every patient received the ideal level of attention from an attending clinician preceptor, in some cases resulting in the preceptor missing certain details in patients’ treatment plans.

Students and patients had different perceptions of some potentially minor consequences of student involvement. For example, students and preceptors worried that students were less efficient, leading to long visits and wait times. Some patients, however, framed long visits positively, appreciating the time students took to ask questions. One patient noted that though long wait times forced her to orient entire days around a visit—or to reschedule because the wait was too long—she still preferred interactions at the SRFC to those at a local FQHC. Another patient tolerated long wait times as a tradeoff for free care (Table 4).

Similarly, patients viewed students’ inexperience in blood draws with understanding, rather than dissatisfaction:

When they have wanted to draw blood and they are a little afraid, I imagine because it is their first time… I say, “It’s okay, calm down, come on, it’s all good,” so that they feel confident, and they can learn. It is so that we can help each other. (Patient)

One student, however, observed that some patients clearly preferred blood draws by non-students, delaying labs until phlebotomists were available.

No interviewed patients reported that student involvement had a strongly negative impact on their health or healthcare experience. A couple of students, however, raised concerns about sensitive situations. One preceptor recalled students using improper techniques when asking patients about traumatic situations, potentially causing distress. At one SRFC, students reportedly did not receive standardized training for performing gynecological exams or introducing themselves as students; rather, clinic leaders trusted students’ discretion based on the clinical experience required to volunteer. A preceptor at the clinic recalled a student overrepresenting their ability level and using improper technique on a patient, forcing the preceptor to intervene mid-examination. One student described the negative consequences of this in the context of an examination viewed as “practice”:

There was a conversation that happened before entering the room between the attending that day and the student, where basically it was decided like, “Well, we don’t really need to do a bimanual, but it’s good practice, so you should do it.” And the patient was in a lot of pain during the procedure and was upset, was physically upset and agitated, and continued to be that way throughout the rest of the visit. And even the Pap procedure with the speculum insertion and removal, the student messed up and she pinched the inside of the patient’s body with the speculum because they removed it in an improper way, which is a mistake that people make sometimes, especially when they’re new to this. But I later found out from the student that she had literally never put a speculum in someone’s body before. She had only practiced it in a lab. And the patient was really upset and in a lot of pain, and a lot of unnecessary pain, and it didn’t seem to be that big of a deal to anyone, I guess. (Student)

### Diverse views on the ethical appropriateness of SRFCs

Patients, students, and preceptors expressed a range of perspectives on the ethical appropriateness of students providing care to patients in the resource-limited settings of SRFCs.

Every patient we interviewed expressed satisfaction or gratefulness. While they expressed frustration with their experiences at other doctors, they frequently viewed the SRFC as a superior, more comfortable alternative:

This is the way it’s done, I think it should be done like that period. Everywhere… If this same setup was at the doctor’s office, more people will go for their physicals when they need to. It would be less of a doctor phobia or, “I don’t want to see that doctor.” (Patient)

Some students and preceptors felt that students and patients benefited equally from the arrangement, believing that SRFCs offered patients high quality services and access to the broader medical system (Table 4).

Other students and preceptors worried that SRFC patients with limited medical options received lower quality care, but nevertheless concluded that SRFCs were ultimately beneficial. Some students, while initially uncomfortable, felt that their SRFC had appropriate safeguards to ensure quality care, such as supervision and a goal of connecting patients with longer-term providers (Table 4). Others viewed SRFCs as a “gray zone,” “lesser of two evils” (student), or form of “harm reduction” (preceptor), because many SRFC patients lack robust healthcare access due to pre-existing social inequities (Table 4). One student framed an ethical question: “Is providing no care better than providing mediocre quality care? Or what’s the threshold for saying it’s ethically better to provide some care than to not provide care at all?” (Student).

Some students, however, expressed unresolved ethical discomfort surrounding students caring for vulnerable patients (Table 4). One student described a friend who only felt comfortable volunteering in roles that “do not exist in a regular hospital or clinic,” such as patient navigation, because she believed that other services offered by the SRFC entailed “actual positions” meant for doctors or social workers. One student expressed the view that the SRFC did not do enough to address underlying issues in the U.S. medical system:

[The SRFC] itself is situated within [the city], for one, which as it’s been mentioned, [the university] could provide free healthcare to everyone in [the city], free, very high-quality healthcare to everyone in [the city] and still be worth billions of dollars… I think sometimes within [the SRFC], we do just reproduce this clinic hospital model that doesn’t work. (Student)

## Discussion

This qualitative study of patients who sought care at two SRFCs on the U.S. East Coast, and students and preceptors who volunteered at these SRFCs, deepens our understanding of the ethics of SRFCs. Study participants identified perceived benefits and disadvantages of SRFCs, and the tradeoffs herein. Overall, all three groups emphasized the value of SRFCs in providing certain hard-to-obtain services for patients facing structural disadvantage, despite limitations in the scope of care, student involvement, and background inequities of the U.S. healthcare system.

At both SRFCs, patients felt that they benefitted by receiving medical care that, in various ways, surpassed the care they had received at other clinics. Patients liked the accessibility of the SRFCs, which offered free or low-cost medical care and often work-friendly hours and location. Patients also valued non-medical services as important contributors to their well-being. No patients we spoke to expressed discomfort with being seen by students. Notably, many preferred interactions with students to those with licensed providers, finding students to be more approachable and attentive.

Students and preceptors recognized these benefits, but also highlighted potential issues at SRFCs. Heterogeneity in students’ preparedness, confidence, and supervision raised concerns about sometimes mismanaged care and painful experiences, particularly for sensitive examinations. Of note, however, both patients and students reported some confidence that good preceptor supervision supported high quality care.

These mixed perspectives both validate and complicate prior ethical analyses of student involvement at SRFCs [21]. Participants in our study highlighted that, while some concerns about students negatively affecting patients’ care are justified, with proper guidance and supervision, students can play a valuable role in delivering vital care through SRFCs. What’s more, students also bring unique optimism and enthusiasm that patients appreciate and benefit from. Bolstered student preparation and processes for evaluating and improving quality could help address these important issues. In particular, SRFCs and their affiliated parent institutions should (1) clarify strict ethical standards of working at the clinic, (2) improve and standardize student screening, preparation, and supervision, (3) ensure adherence to regulations and increase oversight and evaluation, and (4) regularly incorporate patient feedback. More on each of these to follow.

First, SRFCs should provide explicit training on best ethical practices. Clinics should receive patient consent to care from non-licensed providers, as well as center patients’ best interests when offering services—that is, only offer services that are medically indicated for patients, rather than those that may merely be good “practice” for students. Second, SRFCs should strengthen their internal training and supervision to ensure the quality of care provided to patients. This includes rigorously screenings students’ skills and offering more practical training, especially before sensitive exams. Supervision by licensed medical providers is essential for SRFCs to adhere to legal requirements, maintain quality of care, and instill patient confidence. Thus, preceptors should be consistently engaged in patient care and available in ample numbers.

Third, SRFCs should continue to ensure their adherence to regulatory guidelines, though the landscape varies depending on the SRFC location and operating structure. Clinics usually hold licenses under their affiliated university and may be subject to periodic inspection by state regulatory or external review bodies to help verify their safety and quality [25]. For example, at one of the clinics we worked with, a representative from the state’s Department of Public Health has in the past year inspected and confirmed the safety and maintenance of the clinic’s facilities, as well as verified files documenting professional and volunteer responsibilities. This was required for the clinic to maintain the state license under the university with which it is affiliated. The Society of Student Run Free Clinics and affiliated SRFC faculty association offer resources for SRFCs across the U.S. to share best practices and data to improve outcomes, though they do not evaluate individual clinics, and membership is not required. Information on oversight of SRFCs was generally scarce, both in the literature and in our data.

Fourth, and importantly, SRFCs should regularly solicit patient feedback, for example through anonymous patient satisfaction surveys, to help guide their operations and efforts for improvement. At both clinics in this study, some students specifically have quality improvement roles and could be well-positioned to lead these efforts.

In line with other commentators’ experiences [20], the care offered at SRFCs is often constrained by resource limitations: patient factors (e.g. missed appointments), clinic scope (e.g. inability to prescribe medications that require consistent follow-up), capacity (e.g. limited appointment availability), and organization (e.g. inadequate communication with outside providers). The resulting challenges in continuity and scope of care could negatively impact patients’ health—particularly if patients choose SRFCs instead of better-equipped providers, such as FQHCs for complex chronic conditions and emergency rooms for urgent situations. Organizational improvements, such as enhancing coordination with a network of providers, increased recruitment of preceptors, and patient education, could help mitigate these harms.

As others have noted, students benefit from learning at SRFCs [2, 12, 13, 15]. At SRFCs, students are afforded opportunities to practice clinical skills in interprofessional settings, earlier and with greater independence than in many other clinical experiences. Students also gain greater social justice awareness and learn to consider social determinants of health when forming treatment plans. Such lessons could equip students to help not just current SRFC patients, but also future patients in other clinical settings.

Some ethicists fear that SRFCs could negatively impact students’ perceptions of working with underserved populations [21]. Though no interviewed students directly confirmed this effect, some expressed emotional strain as they worked in resource-limited environments with patients in complex and often traumatizing situations. Thus, SRFCs should consider offering additional support, such as formal discussions about emotional challenges or the implications of learning from and caring for patients from vulnerable backgrounds, to avoid deterring students from similar work in the future.

In deliberating about the ethical acceptability of SRFCs, the perceived benefits and harms must be considered within the context of the strained and fragmented U.S. healthcare system. Patients often perceived SRFCs as better than other healthcare options, which many found to be prohibitively expensive, inaccessible, intimidating, or uncomfortable. When patients reported discomfort at SRFCs in some circumstances—such as blood draws and long wait times—they viewed such inconveniences as worthy trade-offs for the free and attentive care they received. Of note, patients at non-SRFC clinics report similar negative experiences, such as improper student supervision [26], long wait times [27], and incorrectly conducted pelvic exams [28]. The reported experiences of these patients and students at SRFCs align with well-documented challenges in the U.S. medical landscape, such as lack of affordable care, widespread inequity in access to and quality of care, and high rates of preventable or treatable deaths, particularly when compared to other high-income countries [29, 30]. Given this, most interviewed students and preceptors believed that despite their limitations, SRFCs offered access to certain kinds of care that patients would not otherwise receive and that helped patients to avoid unnecessary emergency room visits. Some students, however, expressed concerns that SRFCs merely reproduced systemic healthcare injustices.

These perspectives echo ethicists’ complex views on SRFCs as “stopgaps” and the tension between their utility as safety net providers and their potential hindrance of more transformative change [22]. To reconcile these tensions, SRFCs should more clearly define their role within the healthcare landscape, prioritize coordinating care and connecting patients with better resourced providers, and/or refocus on providing non-medical resources. In addition, as mentioned above, SRFCs should strive to (1) clarify ethical standards, (2) improve and standardize student screening, preparation, supervision, and debriefing (3) adhere to regulations and oversight, and (4) regularly solicit and incorporate patient perspectives to both validate and critique their work, Critically, work to improve SRFCs should be accompanied by broader improvements to healthcare access and quality for all.

Our study has several limitations. The patients, students, and preceptors who agreed to be interviewed were more likely to have positive views of the SRFC; for example, we had no access to patients who had had such negative experiences that they chose to stop attending. Additionally, we worked with two well-established SRFCs; however, four other clinics we contacted either did not respond or did not have the infrastructure or policy to support research. SRFCs across the country are likely to vary significantly. One preceptor, for example, noted that the SRFC they supervised was significantly better resourced than one they had volunteered with in medical school. Many SRFC benefits and limitations, which influence their ethical acceptability, are dependent on clinic-specific operations—for example, supervisory structures or the range of patient services offered. The experiences shared by patients, students, and preceptors in our study, and their implications for the ethics of SRFCs, may not be universally applicable to all SRFCs.

## Conclusion

Our study reveals that participants at SRFCs find them to be a valuable, albeit imperfect, way to provide care to patients whose access to comprehensive quality healthcare is limited within the U.S. healthcare system. Patients and students both benefit from their involvement in SRFCs. Patients particularly highlighted SRFCs’ accessibility, non-medical services, and their positive interactions with students, often finding SRFCs preferable to other medical options. Students reported gaining practical skills and valuable knowledge about social justice in medicine. Nevertheless, some students and preceptors identified ethical concerns about the limitations of SRFCs and implications of student involvement, revealing operational changes SRFCs should consider to improve their care. SRFCs’ simultaneous advantages and limitations reveal that, ultimately, improvements to SRFCs should be accompanied by system-level efforts to improve healthcare access and quality for patients facing social and economic marginalization.

## Electronic Supplementary Material

Below is the link to the electronic supplementary material.

Supplementary Material 1
