# Supplementary material for: Views from patients, students, and preceptors about the ethics of student-run free clinics
Source: J Community Health. 2025 Jan 20;50(3):514–26. doi: 10.1007/s10900-024-01438-2 (PMC12069495; doi:10.1007/s10900-024-01438-2)
Supplement: Supplementary file 1 — Supplementary Material 1 [file 10900_2024_1438_MOESM1_ESM.docx]

**Reviewer 1.**

1. The authors identify serious ethical issues in having unsupervised and/or unprepared medical students managing patients in Student-Run Free Clinics. Pleasant interactions with patients are no substitute for competency.

*Response: Thank you for this comment. We appreciated hearing that Reviewer 1’s overall take-away from the paper was that student-run free clinics (SRFCs) can raise serious ethical issues regarding inadequate supervision and unprepared students providing patient care. While this is an important finding of this study, there was variation in how patients, students and preceptors perceived the adequacy of the supervision and the quality of care provided. For example, while some student volunteers felt occasionally unprepared and inadequately supervised in managing patients, many patients and students considered the supervision provided by preceptors as adequate, important to the functioning of these clinics, and appreciated by them (see Table 4, quotes under the subthemes “Students feel well-supervised” and “Patients say they rely on the supervision that students receive and feel comfort knowing that the supervisor is available to them”). The model in both clinics is multilayered, as the least experienced students often provide only non-medical services (ex. translation, social services), or, when offering medical services, see patients under the guidance of more experienced students.* *Students ultimately consult with preceptors about each case (see Table 4, quote under subtheme “Students feel well-supervised”). We have made several edits to better convey the nuances of our findings, especially in the introduction and discussion.*

2. Added to this are inadequate resources which in effect lead to some of these poor patients receiving care that is of inferior quality. One comes away from reading this article sensing that there is an attitude among those involved in these clinics that what they are doing for these patients is better than nothing even if it involves delivering inferior quality care.

*Response: Thank you for these comments. One of the motivations for conducting this study was concern that these clinics were taking advantage of patients who deserve better care. In the end, we came away with a better appreciation for the niche that these clinics fill and the value of, and limitations of, the care that they do provide. Many of the patients who use these clinics would unfortunately have difficulty accessing services provided by these clinics, such as STD screening, blood pressure management, care for minor infections, and the non-medical services. Unfortunately, in the US, access to primary care is challenging (see Blumenthal et al, for e.g.,* [*https://www.commonwealthfund.org/publications/fund-reports/2024/sep/mirror-mirror-2024*](https://www.commonwealthfund.org/publications/fund-reports/2024/sep/mirror-mirror-2024)*), as we now emphasize in the Discussion. In addition, SRFCs can facilitate referrals to other sources of care needed by patients. In the manuscript and in Table 4, we describe the perceived benefits, limitations, and ethical issues associated with SRFCs in more detail (see Table 4, quote under subthemes “SRFC offers patients non-medical services” and “Patients receive high-quality care at SRFC that may otherwise be difficult to access”).*

*Limitations on the scope of care that SRFCs can provide are real, however, and patients do need more care than they get at these clinics; these clinics can and do play a valuable role in bridging care to other providers, as we note in our paper (see Table 4, quote under subtheme “Supervision and referrals to outside providers mitigate any negative impact of student involvement”). Additionally, we note that some of patients’ complaints about SRFCs, such as long wait times or improperly conducted pelvic examinations, are also common at other, non-student-run clinics. While not an excuse for SRFCs, this also reveals more general issues within the U.S. healthcare system.*

*Finally, because some students and preceptors raised concerns about the care provided in certain circumstances (e.g. inexperienced students performing sensitive examinations), we added additional recommendations in our Discussion to highlight the importance of adequate supervision and proper student preparedness.*

3. No mention is made of regular anonymous patient satisfaction surveys being conducted at these clinics. Such outcomes assessments are critical in bringing about inadequacies in these health care facilities. Yet, student satisfaction surveys are regularly carried out in medical schools including the AAMC sponsored GQ survey, the results of which medical schools pay particular attention.

*Response: Thank you for this suggestion, we have added a recommendation for regular anonymous patient satisfaction surveys to the Discussion. We also note that both clinics we worked with have students specifically volunteering in “quality improvement” roles, who may be well-positioned to administer these surveys (One clinic has attempted to do so, though has faced challenges due to patient literacy levels and limited Spanish translator availability).*

4. The use of the identifier Ethical Challenges in the title soft peddles what are actually serious Ethical Issues. Given this, the word challenges should be replaced by issues.

*Response: Thank you for this suggestion, which prompted us to clarify our terminology and to reconsider our title of the paper. As we understand it, both “challenges” and “issues” refer to problems that need to be addressed, and the two terms can be used interchangeably. We now state this in the introduction and use the term “issues” where relevant in the paper, as suggested by the reviewer. Additionally, as our intention with this study was to center the diverse perspectives of those who directly participate in SRFCs about the issues and challenges, we have changed the title of the paper to “Views of Patients, Students, and Preceptors on the Ethics of Student-Run Free Clinics”. We also made several edits in the introduction of the paper to indicate that we report a broad range of views on the perceived benefits, limitations and ethical issues associated with SRFCs.*

5. Throughout the text there is regular confusing use of the words volunteer and student. The word student should be used throughout if that is what is meant.

*Response: Thank you for this helpful comment. We have now explicitly recognized in the introduction that all the students and preceptors were volunteers, and therefore use “student” and “preceptor” throughout the manuscript instead of “volunteer”.*

**Reviewer 2.**

1. The authors have uncovered some serious problems in the student-run free clinics which they surveyed. There are inadequate resources, frequent absence of infrastructures for referral, lapses in the presence of qualified supervisors, medical students attempting to perform well beyond their levels of competency, and other issues. Yet, the authors try to soften their troubling findings by characterizing them as ethical challenges, when in fact they are serious ethical issues. Their current characterization is not acceptable.

*Response: Thank you for these comments, which very much echo comments #1, 2, and 4 by Reviewer 1. Please see our responses to these comments above.*

2. The authors' findings raise the issue of the necessity of periodic evaluation of these clinics much as JACHO inspects hospitals and the RRCs of the ACGME evaluate residency programs. LCME site visits every eight years are unlikely to uncover serious care problems of this nature which involve medical students in student run free clinics. The authors are to be commended for studying these clinics which are both popular and highly lauded, but whose deficiencies are rarely written or spoken about.

*Response: Thank you for this point on the evaluation and oversight of SRFCs. Information on oversight of SRFCs was generally scarce, both in the literature and in our data. Regulatory and licensure requirements for clinics vary according to the state in which they operate (see American Health Lawyers Association,* [*https://www.ama-assn.org/sites/ama-assn.org/files/corp/media-browser/public/ama-foundation/legal-operational-guide-free-medical-clinics.pdf*](https://www.ama-assn.org/sites/ama-assn.org/files/corp/media-browser/public/ama-foundation/legal-operational-guide-free-medical-clinics.pdf)*). For SRFCs in many states, the primary requirement for legal operation is the involvement of licensed providers. Both SRFCs we worked with maintain supervision by licensed preceptors, which, while not a guarantee of quality, may offer at least some level of quality control. To maintain their medical licenses, some clinics are also subject to periodic evaluation by outside bodies. For example, at one of the SRFCs we worked with, publicly available documents show that a representative from the state’s Department of Public Health has in the past year inspected and confirmed the safety and maintenance of the clinic’s facilities, as well as verified files documenting professional and volunteer responsibilities. This was required for the clinic to maintain the state license under the affiliated university.* *Although there is a national society of SRFCs (https://www.studentrunfreeclinics.org/) that creates a network of student- led clinics to share best practices and data in order to improve outcomes, membership is not required and they do not provide evaluations. We have added the limited information that we have about how these SRFCs are evaluated and made a number of recommendations about evaluation and oversight in our Discussion.*

**Reviewer 3.**

1. This is a very important study because it has uncovered and documented the deficiencies and issues affecting the quality of care at student-run free clinics. Corrective action is clearly required not at just these clinics but at many others in the US where similar problems probably exist. Student-run free clinics have generally escaped close evaluation in part because there is currently no national accrediting agency that specifically focuses on them. Quality Improvement Organizations do evaluate the quality of care provided to patients in health care facilities under Medicaid and Medicare, but these clinics would not in general appear on their radar screens.

*Response: Thank you for your comments. We recognize that, though many patients, students, and preceptors felt comfortable with the quality of care offered by SRFCs, there are certainly deficiencies and limitations, and agree that many of these issues are reflective of broader challenges within the U.S. healthcare system—see our response to Reviewer 1’s comment #2. We also recognize that SRFCs in some cases are evaluated by outside organizations, such as the state Department of Public Health, in order to maintain the licenses, they hold under their universities. However, this level of oversight and evaluation may be insufficient—see our response to Reviewer 2’s comment #2. We have also added recommendations for improving training and supervision of students, increasing oversight and evaluation by outside bodies, and adding periodic anonymous patient surveys in our Discussion.*

2. Referring to these serious ethical issues as challenges both in the title and text is really ducking calling a spade a spade. The authors need to correct this.

*Response: Thank you for these comments. Please see our response to Reviewer 1’s comment #4. We recognize the important ethical issues and challenges and are suggesting a modified title.*
